# Blood Pressure Optimization During Fetoscopic Repair of Open Spinal Dysraphism: Insights from Advanced Hemodynamic Monitoring

**DOI:** 10.3390/jcm14228055

**Published:** 2025-11-13

**Authors:** Benjamin Vojnar, Michael Belfort, Caitlin D. Sutton, Corinna Keil, Ivonne Bedei, Gerald Kalmus, Hinnerk Wulf, Siegmund Köhler, Christine Gaik

**Affiliations:** 1 Department of Anesthesiology and Intensive Care Medicine, Faculty of Medicine, Marburg University, Baldingerstrasse, 35033 Marburg, Germany; 2 Department of Anesthesiology and Intensive Care Medicine, University Hospital Giessen and Marburg, Campus Marburg, 35043 Marburg, Germany; 3 Department of Obstetrics and Gynecology, Baylor College of Medicine, Houston, TX 77030, USA; 4 Texas Children’s Fetal Center, Texas Children’s Hospital, Houston, TX 77030, USA; 5 Department of Pediatric Anesthesiology, Perioperative, and Pain Medicine, Texas Children’s Hospital, Baylor College of Medicine, Houston, TX 77030, USA; 6 Department of Obstetrics and Perinatal Medicine, University Hospital of Giessen and Marburg, Campus Marburg, 35043 Marburg, Germany; 7 Department of Prenatal Diagnosis and Fetal Therapy, Justus Liebig University Giessen, 35392 Giessen, Germany

**Keywords:** fetoscopic surgery, open spinal dysraphism, advanced hemodynamic monitoring, fetal heart rate, intraoperative hypotension

## Abstract

**Background/Objectives**: Fetoscopic repair of open spinal dysraphism (OSD) is a rare intrauterine procedure performed in specialized fetal surgery centers. Conducted under restrictive fluid management and continuous tocolysis, it poses substantial challenges to maternal hemodynamic stability. Blood pressure optimization with vasopressor boluses is often required, yet intraoperative hemodynamic data remain limited. **Methods**: This prospective observational study was conducted between December 2023 and January 2025 during fetoscopic repair of OSD at Marburg University Hospital, Germany. Maternal hemodynamics were continuously monitored using pulse contour analysis with the Acumen IQ sensor and HemoSphere platform (Edwards Lifesciences, Irvine, CA, USA). To stabilize arterial pressure, cafedrine/theodrenaline (Akrinor, Ratiopharm, Ulm, Germany) was administered as intravenous boluses. Hemodynamic parameters were analyzed immediately before and after each bolus. Fetal heart rate was assessed as a secondary parameter at predefined intraoperative time points when available. **Results**: A total of 13 patients and 110 vasopressor boluses were analyzed. Reported values reflect median percent changes; parentheses indicate the total range. Following maternal blood pressure optimization, mean arterial pressure increased by 13.7% (5.9–21.6), systemic vascular resistance index by 23.1% (8.3–36.7), and dP/dtmax by 21.7% (6.3–29.9): *p* < 0.001 for all. Cardiac index and stroke volume index decreased by −6.7% (−11.8 to −0.6), *p* < 0.001, and −4.3% (−9.8 to 1.8), *p* = 0.048, respectively. Fetal heart rate remained stable (+0.4% (−0.8 to 1.5); *p* = 0.470). A total of 38 HPI alerts were followed by hypotension, with a median latency of 120 s (80–235); 73 alerts were not followed by hypotension during the observation period. **Conclusions**: Intermittent cafedrine/theodrenaline boluses significantly increased arterial pressure, dP/dtmax, and systemic vascular resistance under conditions of fluid restriction and tocolysis-induced vasodilation. Maternal heart rate remained stable, and cardiac output showed only minor reductions. Fetal heart rate was unchanged following maternal blood pressure treatment, indicating no adverse fetal response to C/T within the observed intraoperative period.

## 1. Introduction

Open spinal dysraphism (OSD), previously referred to as spina bifida aperta, is a congenital, non-lethal central nervous system malformation associated with considerable morbidity [1]. The prevalence in Europe is approximately 5.2 per 10,000 pregnancies, with 1.8 per 10,000 live births [2]. Its pathophysiology follows the “two-hit hypothesis”: the initial defect results from failed neural tube closure, while secondary damage occurs due to mechanical trauma and exposure to neurotoxic amniotic fluid [3,4,5,6]. The primary objective of prenatal OSD repair is to prevent the “second hit” by closing the defect during the second trimester [1,7]. The Management of Myelomeningocele Study (MOMS), the only randomized trial comparing prenatal and postnatal closure, demonstrated significant benefits of prenatal intervention, including improved motor outcomes and reduced need for ventriculoperitoneal shunting. However, the open approach was associated with increased maternal and fetal risks, such as preterm delivery and uterine complications [8]. To mitigate these risks, minimally invasive techniques have been developed in a limited number of specialized fetal surgery centers to reduce maternal and fetal morbidity [9,10,11].

One of these techniques is the laparotomy-assisted fetoscopic approach, also commonly referred to as the hybrid technique [1,9]. Between 2010 and 2020, the International Fetoscopic Neural Tube Defect Repair Consortium reported 300 fetoscopic repairs performed across 14 centers worldwide, predominantly using a laparotomy-assisted (hybrid) approach [11]. The Texas Children’s Fetal Center described 191 hybrid repairs (2014–2024), representing the largest single-center experience to date [12].

The University Hospital of Giessen and Marburg (UKGM) was the first center within the German-speaking area to establish laparotomy-assisted fetoscopic repair of OSD. Following an extensive period of institutional preparation and team training, the center was accepted into the international MMC consortium [5,10,11]. Unlike the open fetal approach involving a hysterotomy, the laparotomy-assisted fetoscopic procedure provides uterine access through three transuterine trocars, avoiding a full-thickness uterine incision and thereby reducing uterine trauma [5,13,14]. This fundamental difference reduces surgical trauma but also affects maternal hemodynamics during surgery.

In contrast to the open fetal approach, the laparotomy-assisted fetoscopic technique uses controlled intrauterine CO_2_ insufflation (10–15 mmHg) to create an intrauterine working space, which may transiently affect maternal venous return and cardiac output, while providing optimal visualization of the fetal lesion [5,13,14,15]. These hemodynamic effects are further influenced by the pharmacological conditions required to maintain uterine relaxation.

Tocolysis during fetal surgery commonly relies on high concentrations of volatile anesthetics, sometimes combined with additional agents such as magnesium sulfate, atosiban, or, less frequently, indomethacin [5,9,14,16]. Volatile anesthetics induce uterine and systemic vasodilation; magnesium sulfate enhances smooth-muscle relaxation through calcium antagonism; atosiban decreases uterine contractility but has rarely been linked to pulmonary capillary leakage; and indomethacin inhibits prostaglandin synthesis [17,18,19]. These combined vasodilatory and permeability-altering effects can increase the risk of maternal pulmonary edema [9,14,15].

Consequently, focused anesthetic management with cautious volume replacement and intermittent vasopressor administration is essential to maintain blood pressure (BP) stability in this challenging setting, counteract systemic vasodilation, and prevent maternal fluid overload and pulmonary edema [14,17]. Blood pressure stabilization during these procedures is typically achieved internationally with vasopressors, most commonly phenylephrine or ephedrine, administered intermittently or by continuous infusion depending on institutional practice [12,20]. In German-speaking countries, however, the fixed-dose combination of cafedrine/theodrenaline (C/T) is a well-established treatment for obstetric hypotension and has also been used in fetal surgery [9,21,22]. At our institution, it serves as the standard vasopressor for intraoperative blood pressure management [9].

Most available data focus on surgical and fetal outcomes, with limited insight into maternal hemodynamics. Therefore, the present study was conducted to explore maternal hemodynamic responses following bolus administration of C/T using pulse contour analysis. By analyzing changes in cardiac index (CI), maximal rate of arterial pressure rise (dP/dtmax), and systemic vascular resistance index (SVRI), we aimed to characterize the dynamic interaction among preload, myocardial contractility, and afterload during blood pressure optimization in this unique physiological setting. In addition, FHR was evaluated during bolus administration as a surrogate marker of uteroplacental perfusion and fetal well-being. These findings may help optimize existing hemodynamic management strategies and support more targeted blood pressure therapy in this complex fetal surgical context. Furthermore, the results provide a framework for comparing the hemodynamic profile of C/T with internationally used vasopressors such as phenylephrine or ephedrine.

## 2. Materials and Methods

### 2.1. The Study Design and Setting

This prospective observational study was approved by the Ethics Committee of Philipps University Marburg on 14 June 2023 (reference: 23-115 BO; Chair: Prof. Carola Seifart). It was registered in the German Clinical Trials Register (DRKS; ID: DRKS00033180) on 6 December 2023, prior to enrolment of the first patient, and conducted at the Department of Anesthesiology and Intensive Care Medicine, Marburg University Hospital, Germany, between December 2023 and January 2025.

**Figure 1:**
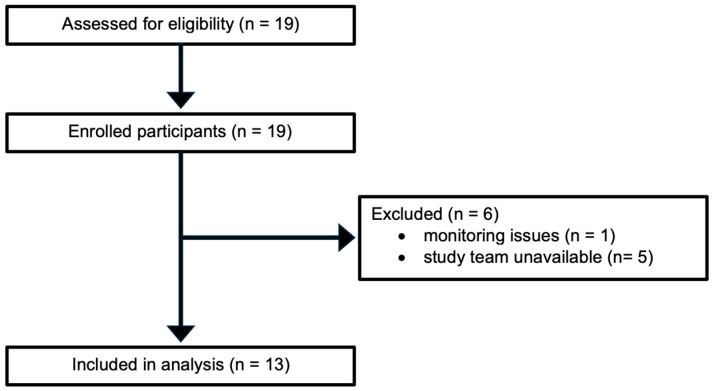
Flow diagram of patient enrolment, exclusion, and analysis.

Patient selection for the surgical procedure was based on established criteria from the MOMS and the International Fetoscopic Neural Tube Defect Repair Consortium [5,9,10,11]. Inclusion criteria comprised a singleton pregnancy with an open spinal lesion between T1 and S1, evidence of hindbrain herniation on fetal MRI, normal fetal karyotype, and gestational age between 19 + 0 and 26 + 0 weeks at the time of surgery. Exclusion criteria included a fetal anomaly unrelated to OSD, severe kyphosis (≥30°), maternal obesity (BMI > 40 kg/m^2^), placenta previa or placental abruption, increased risk of preterm birth, and previous spontaneous singleton delivery prior to 37 weeks, as well as other factors defined in the modified MOMS protocol [5]. For inclusion in this study, patients had to be aged ≥ 18 years and scheduled to undergo laparotomy-assisted fetoscopic repair of OSD at our institution. The enrolment process and reasons for exclusion are illustrated in Figure 1.

Written informed consent was obtained from all patients prior to inclusion. Details of the fetoscopic procedure, including the laparotomy-assisted hybrid technique, were previously published [5,9]. The manuscript adheres to the Strengthening the Reporting of Observational Studies in Epidemiology (STROBE) guidelines.

### 2.2. Advanced Hemodynamic Monitoring

Patients were monitored using the Acumen™ IQ sensor and the HemoSphere platform, version 02.01.000.061 (Edwards Lifesciences, Irvine, CA, USA), which continuously calculate advanced hemodynamic parameters via pulse contour analysis, including arterial blood pressure and the Hypotension Prediction Index (HPI). The HPI is an algorithm-based machine learning model that analyzes arterial pressure waveforms to estimate the likelihood of an impending hypotensive event, defined as a mean arterial pressure (MAP) < 65 mmHg lasting for at least one minute [23,24,25,26].

### 2.3. Management of Intraoperative Hypotension

All patients received C/T (Akrinor™, Ratiopharm, Ulm, Germany) as intravenous boluses to restore MAP. Each ampule contains 200 mg cafedrine and 10 mg theodrenaline. Cafedrine is a synthetic compound formed by covalently linking norephedrine and the xanthine derivative theophylline; theodrenaline results from linking theophylline to norepinephrine [27,28,29]. The blood pressure increase is primarily initiated by α-adrenergic vasoconstriction mediated by the norepinephrine component of theodrenaline, followed by β_1_-adrenoceptor-mediated inotropic stimulation, induced by both norephedrine (from cafedrine) and norepinephrine (from theodrenaline). Theophylline, a phosphodiesterase inhibitor, potentiates this inotropic response by increasing intracellular cAMP. Clinical data confirm a rapid onset, preload recruitment, and increased inotropy as main mechanisms, with minimal chronotropic and moderate vasoconstrictive effects [27,28,30,31]. The bolus dose of C/T was determined by the attending anesthesiologist according to the degree of hypotension and the patient’s hemodynamic response to prior boluses. In our institutional practice, a 40 mg bolus is most commonly used for intraoperative blood pressure optimization, whereas 20 mg is typically administered in cases of mild hypotension and 60 mg in cases of pronounced hypotension. Dosage was reported in terms of cafedrine content only.

### 2.4. Assessment of Hemodynamic Response

Pre-treatment values were defined as the arithmetic mean of all measurements obtained during the 2 min preceding bolus administration, with data acquired at 20 s intervals. To evaluate the hemodynamic response, the mean of the final three values recorded between minutes 2–3 post-administration—corresponding to three consecutive 20 s intervals—was calculated (“post-treatment”). This time window was chosen to reflect stabilized post-intervention conditions following the initial pharmacodynamic effect. Relative changes from baseline were calculated for each parameter.

### 2.5. Fetal Heart Rate

In our study fetal surveillance was provided intraoperatively by a maternal-fetal medicine specialist, who continuously monitored FHR and performed Doppler assessments of the umbilical and middle cerebral arteries as well as the ductus venosus, including calculation of the pulsatility (PI) and resistance indices (RI). To evaluate potential effects of maternal vasopressor administration, all bolus events were screened for available FHR values within a predefined observation window—specifically, 10 min before and 10 min after each bolus. Continuous FHR monitoring was not always feasible throughout the procedure. Only vasopressor boluses with at least one FHR value in both the pre- and post-intervention windows were included. If multiple values were available within a window, the arithmetic mean was used for comparison.

### 2.6. Aim of This Study

The aim of this study was to investigate maternal hemodynamic responses during laparotomy-assisted fetoscopic repair of OSD, with a specific focus on blood pressure stabilization using C/T. The primary outcome was to evaluate alterations in preload, myocardial contractility, and afterload associated with vasopressor administration using advanced hemodynamic monitoring. The secondary outcome, FHR, was monitored to evaluate potential changes in uteroplacental perfusion in response to maternal blood pressure interventions and assessed at predefined intraoperative time points when available. In addition, the extent of intraoperative hypotension was prespecified and quantified by the number and duration of hypotensive episodes and the time-weighted average of MAP <65 mmHg. Finally, the Hypotension Prediction Index (HPI) was examined to explore its potential role in identifying impending hypotensive events during surgery.

### 2.7. Statistical Analysis and Data Management

Descriptive analyses were conducted to summarize absolute values and relative changes in advanced hemodynamic parameters following vasopressor administration, the extent of intraoperative hypotension, and potential alterations in FHR. The distribution of continuous parameters was assessed using Shapiro–Wilk test. As data were not normally distributed, paired comparisons before and after maternal blood pressure optimization were performed using Wilcoxon signed-rank test. Results are reported as median differences with interquartile ranges (IQRs). A two-sided *p*-value < 0.05 was considered statistically significant. All analyses were performed using IBM SPSS Statistics for Mac, version 30.0 (IBM Corp., Armonk, NY, USA), Acumen Analytics, version 2.0.0 (Edwards Lifesciences, Irvine, CA, USA) and Microsoft Excel for Mac, version 16.92 (Microsoft Corp., Redmond, WA, USA). The target sample size was guided by case numbers from similar studies and aligned with the expected number of procedures within our defined observation period for this rare procedure [32,33].

## 3. Results

### 3.1. Maternal Demographic and Clinical Characteristics

Thirteen patients were included in the analysis. The mean age was 31 ± 6 years, mean body weight 75 ± 20 kg, mean height 163 ± 5 cm and mean gestational age 24 + 3 weeks. The mean estimated fetal weight was 629 ± 87 g.

All patients were in good general health. Two of the 13 patients had pre-existing arterial hypertension; one of them was receiving methyldopa 250 mg preoperatively. No gestational hypertensive disorders were documented, and no additional chronic cardiovascular disease or long-term cardiovascular medication was reported.

### 3.2. Anesthetic Management

Baseline hemodynamic parameters were recorded after a standardized 5 min resting period. The median heart rate (HR) was 85 beats per minute (bpm) (IQR: 82–92), and the MAP was 87 mmHg (IQR: 78–96). The median cardiac index (CI) was 3.6 L·min^−1^·m^−2^ (IQR: 3.2–4.2) and the median SVRI was 1859 dyn·s·cm^−5^·m^2^ (IQR: 1499–2126). A passive leg raise test was performed in all patients, to assess preload status prior to surgery. Resulting in a median relative change in stroke volume index (SVI) of +7.4% (IQR: 5–12). Median MAP increased from 85 mmHg (IQR: 74–89) to 89 mmHg (IQR: 84–99).

All patients received an epidural catheter placed between the T11/T12 and L3/L4 interspaces. The initial epidural bolus was administered toward the end of surgery and consisted of a median 12 mL of 0.2% ropivacaine (IQR: 10–12) combined with a median 10 µg sufentanil (IQR: 10–10). Postoperative analgesia was provided via patient-controlled epidural analgesia (PCEA) using 0.2% ropivacaine with 0.75 µg/mL sufentanil. The basal infusion rate was 6 mL/h, with patient-controlled boluses of 4 mL and a 30 min lockout interval.

General anesthesia was induced with fentanyl, propofol, and rocuronium. Median induction doses were 0.3 mg fentanyl (IQR: 0.3–0.4), 200 mg propofol (IQR: 150–200), and 70 mg rocuronium (IQR: 50–80). Details of intraoperative medication are shown in Table 1.

A total of 110 vasopressor boluses were administered intraoperatively, yielding a cumulative cafedrine dose of 4200 mg. The median bolus dose was 40 mg (IQR: 40–40), with individual doses ranging from 20 mg to 60 mg. Most boluses (83.6%, *n* = 92) were 40 mg, followed by 20 mg in 12.7% (*n* = 14) and 60 mg in 3.6% (*n* = 4). Hemodynamic values before and after blood pressure optimization, along with relative changes, are summarized in Table 2.

Due to the observational design and absence of a standardized protocol aligned with bolus timing, complete paired FHR data were available for 34 boluses. These were analyzed to compare FHR values before and after maternal blood pressure optimization. Absolute and relative changes are presented in Table 2.

A total of 38 HPI alerts were followed by hypotensive episodes, with a median time to hypotension of 120 s (IQR 80–235). In 73 instances, the same alert criteria were met without subsequent hypotension during the observation period. Table 3 summarizes cumulative and per-patient data on intraoperative monitoring time and hypotension. As a surrogate marker of tissue perfusion, maternal lactate concentrations were analyzed, increasing from a median of 1.45 mmol/L (IQR 1.15–1.63) preoperatively to 2.20 mmol/L (IQR 1.50–3.00) postoperatively.

## 4. Discussion

Blood pressure optimization with C/T significantly increased mean arterial pressure, dP/dtmax, and systemic vascular resistance under fluid restriction and continuous tocolysis-induced vasodilation. Low dynamic preload indices (SVV, PPV) indicated hypotension without hypovolemia, supporting the vasopressor-first approach adopted at many centers to minimize pulmonary edema risk. Fetal heart rate remained stable following maternal blood pressure treatment, suggesting that C/T did not adversely affect fetal heart rate through maternal hemodynamic alterations within the observed intraoperative period.

Uterine relaxation was maintained with sevoflurane, atosiban, and magnesium sulfate, all of which may affect maternal hemodynamic stability [5,9]. Sevoflurane was administered according to institutional protocol, with concentrations temporarily increased up to 2 MAC to achieve profound uterine relaxation during periods of uterine manipulation [5,9]. Several studies have shown that sevoflurane decreases systemic vascular resistance and mean arterial pressure, often requiring vasopressor support [14]. Sevoflurane, like other volatile anesthetics, crosses the placenta by passive diffusion and rapidly equilibrates between maternal and fetal compartments, resulting in measurable fetal exposure during maternal general anesthesia [20].

Atosiban, a selective oxytocin and vasopressin V1A receptor antagonist, is used to inhibit uterine contractions and maintain uterine relaxation during fetal surgery [34,35]. It provides effective tocolysis without relevant maternal hemodynamic side effects, showing no clinically significant influence on heart rate or blood pressure. Placental passage of Atosiban is minimal, with umbilical vein concentrations markedly lower than maternal plasma levels, indicating limited fetal exposure [36].

Magnesium sulfate was administered as an adjunct tocolytic to promote uterine relaxation and reduce the required concentration of volatile anesthetics during fetoscopic repair [12,37]. It produces peripheral and uterine vasodilation by inhibiting calcium entry through voltage-dependent calcium channels in vascular smooth muscle, leading to decreased vascular tone and systemic blood pressure [38]. Magnesium freely crosses the placenta by passive diffusion, resulting in fetal serum concentrations that closely reflect maternal levels [38].

In our cohort, this induced decrease in vascular tone appeared to reverse following C/T bolus administration, as reflected by a significant increase in SVRI. This finding is consistent with the α-adrenergic vasoconstrictive mechanism previously described for C/T, primarily mediated by the norepinephrine component of theodrenaline [28,29,30]. The observed increase in SVRI was accompanied by a significant rise in mean arterial pressure, indicating effective restoration of systemic afterload in a vasodilatory setting.

Direct comparison of these results with other vasopressors is challenging, as hemodynamic data during laparotomy-assisted fetoscopic repair of open spinal dysraphism are extremely limited [5,10]. As both fetoscopic fetal surgery and cesarean delivery under spinal anesthesia are associated with vasodilation and reduced systemic vascular resistance, hemodynamic data from obstetric anesthesia may offer a physiologic context for interpretation of the current results. Internationally, vasopressors such as phenylephrine and ephedrine are commonly used in obstetric anesthesia and during fetal surgery for OSD repair [20,39].

With regard to placental transfer, ephedrine freely crosses the placenta, leading to fetal catecholamine and lactate increases [40], whereas phenylephrine shows minimal transplacental passage and is associated with a more stable fetal acid-base status [39,40]. For C/T, no evidence on placental transfer or direct fetal exposure is currently available.

Phenylephrine restores arterial pressure primarily through α_1_-adrenergic vasoconstriction, resulting in increased systemic vascular resistance and mean arterial pressure, but often at the expense of reduced cardiac output [41]. In contrast, C/T in our study produced a more balanced hemodynamic response, characterized by a marked increase in systemic vascular resistance and arterial pressure, accompanied by only modest reductions in cardiac and stroke volume indices. This profile may indicate effective restoration of afterload and vascular tone without relevant impairment of cardiac output, which would be consistent with the combined α- and β-adrenergic activity of C/T [27,28,30,31].

Ephedrine acts primarily through β-adrenergic stimulation and endogenous norepinephrine release, producing greater increases in cardiac output and heart rate with less pronounced effects on vascular resistance [42,43]. In our cohort, C/T was associated with higher vascular resistance and arterial pressure without altering heart rate, reflecting balanced adrenergic activity. As previously noted, direct comparison of hemodynamic responses to vasopressor administration between obstetric anesthesia and fetoscopic OSD repair should be made with caution; however, the present findings may provide a useful framework for comparing the pharmacodynamic profiles of different agents in these related settings.

Despite restrictive fluid administration, indicators of potential hypovolemia—such as stroke volume variation (SVV), pulse pressure variation (PPV), and perioperative urine output—remained within physiological limits prior to hypotension management. Before blood pressure optimization, both SVV and PPV were below the 12% threshold, suggesting that the observed hypotension occurred without volume responsiveness and was therefore unlikely due to hypovolemia [44,45]. This observation supports the rationale for maintaining a restrictive fluid strategy and is an important consideration in preventing maternal pulmonary edema [46,47]. In such therapeutic decisions, advanced hemodynamic monitoring can be particularly valuable, as it enables goal-directed therapy by distinguishing whether hypotension is driven by reduced preload, impaired contractility, or decreased vascular tone, thereby avoiding empiric fluid loading and guiding individualized hemodynamic support [48].

Pulse contour analysis with the Acumen™ IQ sensor provides a continuous, minimally invasive estimate of left ventricular contractility via dP/dtmax calculated from the steepest slope of the systolic upstroke of the arterial pressure curve. Abdallah et al. showed that vasopressors like phenylephrine and ephedrine significantly increased dP/dtmax, supporting its sensitivity to inotropic changes and its value as a surrogate marker under dynamic hemodynamic conditions [43]. Despite a significant increase in dP/dtmax following C/T administration, both SVI and CI declined slightly but significantly. While increased inotropy would typically be expected to augment stroke volume, the concurrent rise in SVRI suggests elevated afterload as a contributing factor. In this context, dP/dtmax may predominantly reflect increased ventricular workload rather than a true improvement in contractile function. The combination of non-volume responsiveness and increased afterload may thus explain the paradoxical decrease in CI despite improved contractility. The stable maternal heart rate supports the interpretation that this reduction in CI was primarily driven by changes in stroke volume rather than chronotropic effects. These findings underscore the complex interplay between preload, afterload, and contractility, particularly under the influence of sympathomimetic vasopressors.

Unlike in adults, the fetal response to hypoxemia typically involves heart rate deceleration, which may progress to bradycardia (FHR < 110 bpm) [49,50]. Fetal cardiac output depends largely on heart rate, as the myocardium has limited capacity to increase stroke volume due to structural immaturity and a reduced preload reserve [49,51,52]. Even slight reductions in FHR can significantly impair cardiac output [20]. While bradycardia is a known risk, sustained tachycardia (FHR >160 bpm) can likewise reduce output by limiting ventricular filling and stroke volume [53]. Both extremes, therefore, represent clinically relevant threats to fetal hemodynamic stability.

In this observational setting, FHR did not change significantly at any of the 34 recorded time points before and after maternal hemodynamic stabilization with C/T. These findings suggest that the maternal hemodynamic changes induced by C/T do not adversely affect fetal heart rate during fetoscopic OSD repair, at least within the observed intraoperative timeframe. While previous studies have demonstrated favorable neonatal acid-base status following C/T administration in cesarean delivery, to our knowledge, this appears to be the first report specifically addressing FHR stability in the context of fetoscopic OSD repair. However, given the small sample size (*n* = 34) and the rarity of this surgical indication, these findings should be interpreted with caution.

HPI is a machine learning–based algorithm that continuously evaluates beat-to-beat changes in high-fidelity arterial pressure waveforms to estimate the likelihood of impending hypotension, defined as a mean arterial pressure below 65 mmHg [23,24,25,26]. In the present study, HPI frequently identified gradual declines in arterial pressure several minutes before the mean arterial pressure dropped below this threshold. A comparable temporal relationship has been reported in obstetric anesthesia, with HPI alerts preceding hypotension by approximately 2–3 min [54]. However, approximately two-thirds of all alerts were not followed by hypotension, which underscores the need to interpret HPI in conjunction with additional clinical and hemodynamic indicators. Alerts classified as false positives were not followed by hypotension despite the absence of any active blood pressure management during the observation period. Our study design and the absence of standardized response protocols to HPI alerts represent limitations in interpreting these findings.

Given the limited number of patients and the rarity of fetoscopic OSD repair, future studies should aim to include larger patient cohorts through multicenter collaboration. To systematically evaluate hemodynamic responses to vasopressor administration, a standardized study protocol with predefined maternal blood pressure thresholds is essential to reduce variability and improve comparability across centers. Particular attention should be given to the structured assessment of fetal heart rate before and after vasopressor administration to more accurately characterize potential fetal effects. Furthermore, randomized controlled trials comparing different vasopressor agents using fixed bolus dosing protocols may help identify the most effective and safest strategy for maintaining maternal hemodynamic stability during fetoscopic procedures.

### Strengths and Limitations

Strengths of this study include the systematic evaluation of 110 maternal vasopressor boluses using continuous advanced hemodynamic monitoring, as well as the first-time analysis of FHR in direct temporal association with maternal blood pressure treatment during fetoscopic repair of OSD. However, several limitations must be acknowledged.

First, the relatively small sample size and the single-center design limit the generalizability of the findings.

Second, due to the observational design, the study does not permit hypothesis testing or causal inference. The results should therefore be interpreted as exploratory and hypothesis-generating.

Third, the analysis of FHR was limited to a small subgroup (*n* = 34) with available paired measurements. To the extent possible, we aim for frequent or continuous observation of fetal heart rate, but a specific measurement is not always obtained, limiting paired data availability. As the timing of these measurements was not standardized and may have been influenced by clinical factors, a potential selection bias cannot be excluded.

Fourth, the use of non-standardized bolus doses of C/T may have introduced variability in hemodynamic responses, limiting the comparability across blood pressure treatments. Fifth, only C/T was used for vasopressor therapy, reflecting local clinical standards and potentially limiting international comparability.

Sixth, the results must be interpreted in light of the unique physiological conditions of mid-gestation fetal surgery, for which validated reference ranges for advanced maternal hemodynamic parameters are currently lacking.

Finally, although HPI was included in the analysis, the retrospective nature of the study and the absence of protocolized responses to HPI alerts limit conclusions regarding its predictive accuracy or clinical utility in this setting.

## 5. Conclusions

This prospective observational study characterized maternal hemodynamic responses to intermittent cafedrine/theodrenaline boluses administered for blood pressure stabilization during laparotomy-assisted fetoscopic repair of OSD. C/T effectively restored arterial pressure and systemic vascular resistance under conditions of fluid restriction and continuous tocolysis-induced vasodilation. Low dynamic preload indices (SVV, PPV) confirmed that hypotension occurred in the absence of volume responsiveness, supporting a vasopressor-first approach. Fetal heart rate remained stable following maternal blood pressure therapy, indicating that C/T did not adversely affect fetal heart rate through maternal hemodynamic changes within the observed intraoperative period. These findings expand the limited evidence on maternal–fetal hemodynamics in fetal surgery. Future prospective, multicenter investigations are needed in this high-risk population, particularly those comparing the hemodynamic effects of internationally used vasopressors such as phenylephrine and ephedrine.

## Figures and Tables

**Table 1:** **Intraoperative medication, vasopressor therapy, uterine relaxation, intravenous fluids, and urine output.** The table displays cumulative intraoperative doses per patient for each medication. Bolus dosing of cafedrine/theodrenaline is also presented. The number of observations (*n*) varies because not all patients received each medication. Units and n-values are indicated for each parameter.

Parameter	Median (IQR)
**Anesthesia**	
Sevoflurane, end-expiratory, *n* = 13 [Vol%]	2.5 (2.1–2.8)
Remifentanil, *n*= 13 [µg]	2990 (2290–4536)
**Vasopressor therapy**	
Norepinephrine infusion, *n* = 3 [µg]	1530 (270–2490)
Cafedrine/theodrenaline bolus, *n* = 110 [mg]	40 (40–40)
**Tocolytic therapy**	
Atosiban, *n* = 13 [mg/h]	137 (63–187)
Magnesium sulfate, *n* = 10 [g/h]	3.5 (2.6–3.8)
**Volume therapy**	
Human albumin 5%, *n* = 13 [mL]	750 (500–1000)
Ringer’s acetate, *n* = 6 [mL]	175 (150–350)
Urine output, *n* = 13 [mL]	1600 (1200–1850)

**Table 2:** **Hemodynamic parameters before and after maternal vasopressor administration.** Data are shown as median [IQR]. Percentage changes refer to paired measurements before and after bolus administration of cafedrine/theodrenaline (*n* = 110). Positive values indicate an increase, negative values a decrease. Due to artifacts or missing data, the number of paired measurements varies. Fetal heart rate was available in 34 cases. Statistical significance was assessed using the Wilcoxon signed-rank test; *p*-values < 0.05 were considered significant.

Parameter	*n*	Prior Hypotension Management	After Hypotension Management	Percent Change	*p*-Value
Cardiac Index [L/min/m^2^]	110	3.6 [2.8–4.4]	3.4 [2.7–4.0]	−6.7 [−11.8–−0.6]	<0.001
Stroke Volume Index [mL/m^2^]	110	46 [38–54]	43 [38–51]	−4.3 [−9.8–1.8]	0.048
Maternal Pulse Rate [bpm]	110	78 [73–83]	78 [71–85]	−0.5 [−4.0–1.6]	0.670
Systolic Pressure [mmHg]	110	101 [95–108]	114 [107–125]	13.9 [5.8–20.5]	<0.001
Diastolic Pressure [mmHg]	110	56 [51–60]	63 [55–68]	12.0 [4.5–18.7]	<0.001
Mean Arterial Pressure [mmHg]	110	70 [65–75]	78 [70–86]	13.7 [5.9–21.6]	<0.001
Systemic Vascular Resistance Index [dyn·s·cm^−5^·m^2^]	105	1420 [1139–1760.9]	1670 [1473–1999]	23.1 [8.3–36.7]	<0.001
Stroke Volume Variation [%]	110	6.7 [5.6–8.0]	6.0 [4.3–8.0]	−11.9 [−25.2–−3.7]	0.014
Pulse Pressure Variation [%]	110	8.1 [6.8–10.3]	7.0 [5.7–9.3]	−12.7 [−25.2–0.0]	0.006
Rate of arterial pressure rise [mmHg/s]	110	745 [620–841]	886 [705–1000]	21.7 [6.3–29.9]	<0.001
Eadyn (PPV/SVV)	110	1.3 [1.1–1.4]	1.3 [1.1–1.5]	1.2 [−9.4–15.2]	0.852
Fetal Heart Rate [bpm]	34	128 [121–133]	128 [119–136]	0.4 [−0.8–1.5]	0.470

PPV = pulse pressure variation; SVV = stroke volume variation.

**Table 3:** **Monitoring time and hypotension metrics.** Cumulative and per-patient data on intraoperative hypotension are shown, including the number, duration, and time-weighted average of episodes with MAP < 65 mmHg, as well as mean MAP during hypotension. Values are presented as mean ± SD unless otherwise stated. Calculations were performed using Acumen Analytics (Edwards Lifesciences, Irvine, CA, USA).

Hypotension Metrics	Value
Total monitoring time of the cohort	5350.67 min
Monitoring time per patient	411.59 ± 32.93 min
Number of patients with hypotension	8 of 13 (61.54%)
Total number of hypotensive events	96 events
Average number of hypotensive events per patient	7.38 ± 9.29
Average duration of each hypotensive event	6.39 ± 10.73 min
Mean MAP under 65 mmHg per patient	58.27 ± 13.07 mmHg
Time-weighted average of area under threshold (MAP < 65 mmHg) per patient	0.44 ± 0.95 mmHg

## Data Availability

The datasets generated and analyzed during the current study are not publicly available due to institutional and data protection policies but are available from the corresponding author upon reasonable request.

## Abbreviations

The following abbreviations are used in this manuscript:

bpm	beats per minute
C/T	cafedrine/theodrenaline
CI	cardiac index
CO	cardiac output
dP/dtmax	maximal rate of arterial pressure rise
FHR	fetal heart rate
HPI	Hypotension Prediction Index
HR	heart rate
IQR	interquartile range
MAP	mean arterial pressure
OSD	open spinal dysraphism
PPV	pulse pressure variation
PR	pulse rate
SVI	stroke volume index
SVRI	systemic vascular resistance index
SVV	stroke volume variation
TWA	time-weighted average
